# Analysis of trends in the burden of colorectal cancer in China and globally from 1990 to 2021 with projections for the next 15 years: a cross-sectional study based on the GBD database

**DOI:** 10.3389/fpubh.2025.1518536

**Published:** 2025-05-26

**Authors:** Yulai Yin, Xiaoyu Zhang

**Affiliations:** ^1^Cangzhou Central Hospital Affiliated to Hebei Medical University, Cangzhou, China; ^2^Department of Thyroid and Breast Surgery, Cangzhou Central Hospital, Cangzhou, China

**Keywords:** colorectal cancer, disease burden, incidence rate, prevalence rate, prevention

## Abstract

**Objective:**

To analyze the changes in the burden of colorectal cancer in China and globally from 1990 to 2021, and to explore the disease burden across different age groups and sexes by integrating projected data from 2022 to 2036. This study aims to provide a scientific foundation for formulating targeted prevention and control strategies.

**Materials and methods:**

This study utilized colorectal cancer data from the Global Burden of Disease (GBD) database for the period 1990–2021. Trend analysis was conducted using Joinpoint regression, and future burden projections from 2022 to 2036 were made with an Auto-Regressive Integrated Moving Average (ARIMA) model. Key indicators analyzed included the Age-Standardized Incidence Rate (ASIR), Age-Standardized Prevalence Rate (ASPR), Age-Standardized Mortality Rate (ASMR), and Disability-Adjusted Life Years (DALYs).

**Results:**

Between 1990 and 2021, the burden of colorectal cancer increased both in China and globally, although notable differences were observed across gender and regions. In Chinese men, the ASIR and ASMR have shown a continuous rise, reaching approximately 50 and 300 per 100,000, respectively, by 2021. Projections indicate that these rates will continue to increase through at least 2036. The ASIR in Chinese women also exhibits an upward trend, whereas the global ASIR for women has declined. From 1990 to 2021, both China and the world experienced a steady rise in ASPR, with minimal fluctuations. In contrast, while the ASDR has decreased in both China and globally, the volatility in China’s ASDR is notably more pronounced than that observed worldwide.

**Conclusion:**

The burden of colorectal cancer in China is projected to increase significantly in the coming years, particularly among males and the older adult population. This study provides critical scientific insights for the development of targeted prevention strategies and resource allocation, underscoring the urgent need to enhance early screening initiatives and health education efforts in China.

## 1 Introduction

Colorectal cancer (CRC) represents one of the most critical global public health challenges, characterized by a complex pathogenesis involving both genetic susceptibility and environmental factors. Studies have shown that detrimental dietary habits, lack of physical exercise, obesity, and smoking significantly heighten the risk of CRC (1, 2). Over recent decades, the incidence of CRC has exhibited an alarming upward trend, particularly in rapidly industrializing nations (3). According to the latest statistics, CRC has become the third most common and the second deadliest malignant tumor worldwide (4, 5). In 2020 alone, over 1.9 million new cases and nearly 935,000 deaths were reported globally, underscoring the substantial burden CRC imposes on health systems.

In China, the burden of CRC shows notable regional disparities closely associated with population aging and socioeconomic transitions. Nationally, CRC incidence and mortality have sharply risen over the past 30 years, especially in urban areas, where exposure to risk factors increasingly mirrors that of Western countries (6). Although rural CRC incidence has historically been lower, it has also shown a gradual increase in recent years, which is believed to relate to shifts in lifestyle and dietary patterns. These urban–rural differences highlight imbalances in healthcare access, lifestyle factors, and preventive measures, underscoring an urgent need for targeted interventions to curb CRC incidence and improve health outcomes across regions.

The Global Burden of Disease (GBD) database (7–9) currently provides essential resources for quantifying and understanding epidemiological patterns of CRC, offering data on incidence, mortality, and DALYs across various regions and time points. However, although GBD-based analyses yield valuable insights into global and regional CRC trends, localized longitudinal studies addressing rapid urbanization and population aging—especially in China—remain limited. Furthermore, predictive data on CRC burden (10–12), critical for healthcare resource planning, are sparse, highlighting the need for prospective research to support policy-making and resource allocation. This study undertakes a comprehensive analysis of the CRC disease burden trends in China and globally from 1990 to 2021 based on GBD data, along with projections for the next 15 years. By examining trend analyses and forecasting future burden, this study aims to deliver an evolving landscape of CRC, with implications for health policy, resource prioritization, and preventive strategy development.

## 2 Materials and methods

### 2.1 Data source

The Global Burden of Disease (GBD) study, led by the Institute for Health Metrics and Evaluation (IHME), provides comprehensive and comparable global health metrics. Data for this study were drawn from the GBD 2021 dataset,^1^ which offers key insights into mortality, disease burden, and risk factors across different regions and populations. This dataset is an integrated database documenting incidence, prevalence, and mortality rates of 369 diseases and injuries across 204 countries and territories, stratified by age and sex. For this analysis, data on incidence, prevalence, mortality, and DALYs of CRC for all age groups and both sexes, adjusted for age, were extracted for China and globally from 1990 to 2021.

### 2.2 Statistical analysis

Data on CRC incidence, prevalence, mortality, DALYs, ASIR, ASPR, ASMR, and ASDR, along with crude incidence rate (CIR), crude prevalence rate (CPR), crude mortality rate (CMR), and crude DALY rate (CDR) across age groups, were extracted from the GBD database for both China and global populations. Average annual percentage change (AAPC) and corresponding 95% confidence intervals (95% CI) were calculated using Joinpoint software (National Cancer Institute, Rockville, MD, USA) to assess disease burden trends. Log-transformed age-standardized indicators were fitted into a regression model, specified as ln(y) = *α* + *β*x + *ε*, where y represents the respective age-standardized indicator, and x denotes the calendar year. AAPC was calculated as 100 × (exp(β) − 1), with 95% CI derivable from the model. An AAPC estimate with a 95% CI > 0 indicates an upward trend; < 0 denotes a downward trend; and a CI containing 0 reflects a stable trend.

For future trend projections, the Auto-Regressive Integrated Moving Average (ARIMA) model (13–15) was selected. ARIMA is a widely used model for time series analysis, suitable for predicting future trends based on known historical data. Model parameters were determined through autocorrelation and partial autocorrelation function analysis, with parameter optimization based on the Akaike Information Criterion (AIC) and Bayesian Information Criterion (BIC). Upon training completion, the ARIMA model was applied to forecast CRC incidence, mortality, and DALYs for the next 15 years, providing quantitative results for future burden projections. All ARIMA model prediction results include 95% confidence intervals (95% CI) to account for uncertainty in the forecasted trends. The specific calculation method is as follows: the standard error of the predicted values is calculated based on the standard error of the model residuals, and the 95% confidence interval is computed using the t-distribution: predicted value ± t (0.025, df) × standard error, where t (0.025, df) represents the critical value of the t-distribution with degrees of freedom df at a significance level of 0.025.

Statistical analyses and visualizations were performed using R software (version 4.3.2) and Joinpoint software (version 4.9.1.0) (16–20). A *p*-value <0.05 was considered statistically significant.

### 2.3 Data disclosure statement

Data used in this study were sourced from the GBD 2021 dataset (see text footnote 1), which contains no personally identifiable information. Ethical approval was obtained for the original studies related to this dataset, rendering separate ethical approval for this study unnecessary.

## 3 Results

### 3.1 Descriptive analysis of the burden of colorectal cancer in China and globally

Between 1990 and 2021, the incidence, prevalence, mortality, and DALYs for colorectal cancer (CRC) exhibited distinct trends in China and globally. Overall, the burden of CRC in China showed a more pronounced increase, whereas global trends remained relatively stable. Regarding incidence rates, the number of CRC cases in China rose from 158,389 in 1990 to 658,321 in 2021, with an ASIR increasing from 19.042 per 100,000 people to 31.444 per 100,000, reflecting an average annual percentage change (AAPC) of 1.7% (95% CI: 1.4–1.9%). In contrast, global CRC cases rose from 916,584 to 2,194,143, with a modest ASIR increase from 24.04 per 100,000 to 25.607 per 100,000, yielding an AAPC of 0.2% (95% CI: 0.2–0.2%). In terms of prevalence, CRC cases in China increased from 635,609 in 1990 to 3,605,686 in 2021, with the ASPR rising from 69.9 per 100,000 to 168.62 per 100,000, resulting in an AAPC of 3% (95% CI: 2.8–3.1%). Globally, prevalence increased from 4,266,770 to 11,679,120 cases, with the ASPR rising from 108.25 per 100,000 to 134.837 per 100,000, and an AAPC of 0.7% (95% CI: 0.7–0.8%). For mortality and DALYs, both China and the global figures showed a downward trend. In China, the CRC mortality rate declined from 15.493 per 100,000 in 1990 to 13.637 per 100,000 in 2021, while DALYs decreased from 390.63 per 100,000 to 331.728 per 100,000. Globally, mortality dropped from 15.562 per 100,000 to 12.398 per 100,000, and DALYs fell from 357.326 per 100,000 to 283.242 per 100,000 (Table 1).

**Table 1:** Total cases, age-standardized incidence, prevalence, mortality, and DALY rates of colorectal cancer in China and globally in 1990 and 2021, with corresponding AAPC.

Location	Measure	1990		2021		1990–2021 AAPC
		All-ages cases	Age-standardized rates per 100,000 people	All-ages cases	Age-standardized rates per 100,000 people	
		*n* (95%CI)	*n* (95%CI)	*n* (95%CI)	*n* (95%CI)	*n* (95%CI)
China	Incidence	158,389 (135419–182,577)	19.042 (16.462–21.813)	658,321 (531995–798,063)	31.444 (25.526–37.973)	1.7 (1.4–1.9)
	Prevalence	635,609 (548090–729,557)	69.9 (60.617–79.835)	3,605,686 (2912081–4,349,689)	168.62 (136.582–203.054)	3 (2.8–3.1)
	Deaths	119,303 (102706–137,153)	15.493 (13.429–17.704)	275,129 (223379–330,960)	13.637 (11.088–16.31)	−0.4 (−0.6 - -0.2)
	DALYS	3,565,196 (3027610–4,106,701)	390.63 (333.239–448.924)	6,848,390 (5513407–8,284,228)	331.728 (267.778–400.698)	−0.5 (−0.7 - -0.4)
Global	Incidence	916,584 (866238–951,895)	24.04 (22.544–25.011)	2,194,143 (2001272–2,359,390)	25.607 (23.322–27.516)	0.2 (0.2–0.2)
	Prevalence	4,266,770 (4085520–4,447,809)	108.25 (103.293–112.746)	11,679,120 (10774527–12,538,400)	134.837 (124.213–144.77)	0.7 (0.7–0.8)
	Deaths	570,319 (536545–597,669)	15.562 (14.487–16.313)	1,044,072 (950188–1,120,169)	12.398 (11.241–13.306)	−0.7 (−0.8 - -0.6)
	DALYS	14,396,658 (13568749–15,166,576)	357.326 (336.623–375.739)	24,401,100 (22689369–26,161,518)	283.242 (263.114–303.326)	−0.7 (−0.8 - -0.6)

### 3.2 Joinpoint regression analysis of colorectal cancer burden in China and globally

**Figure 1:**
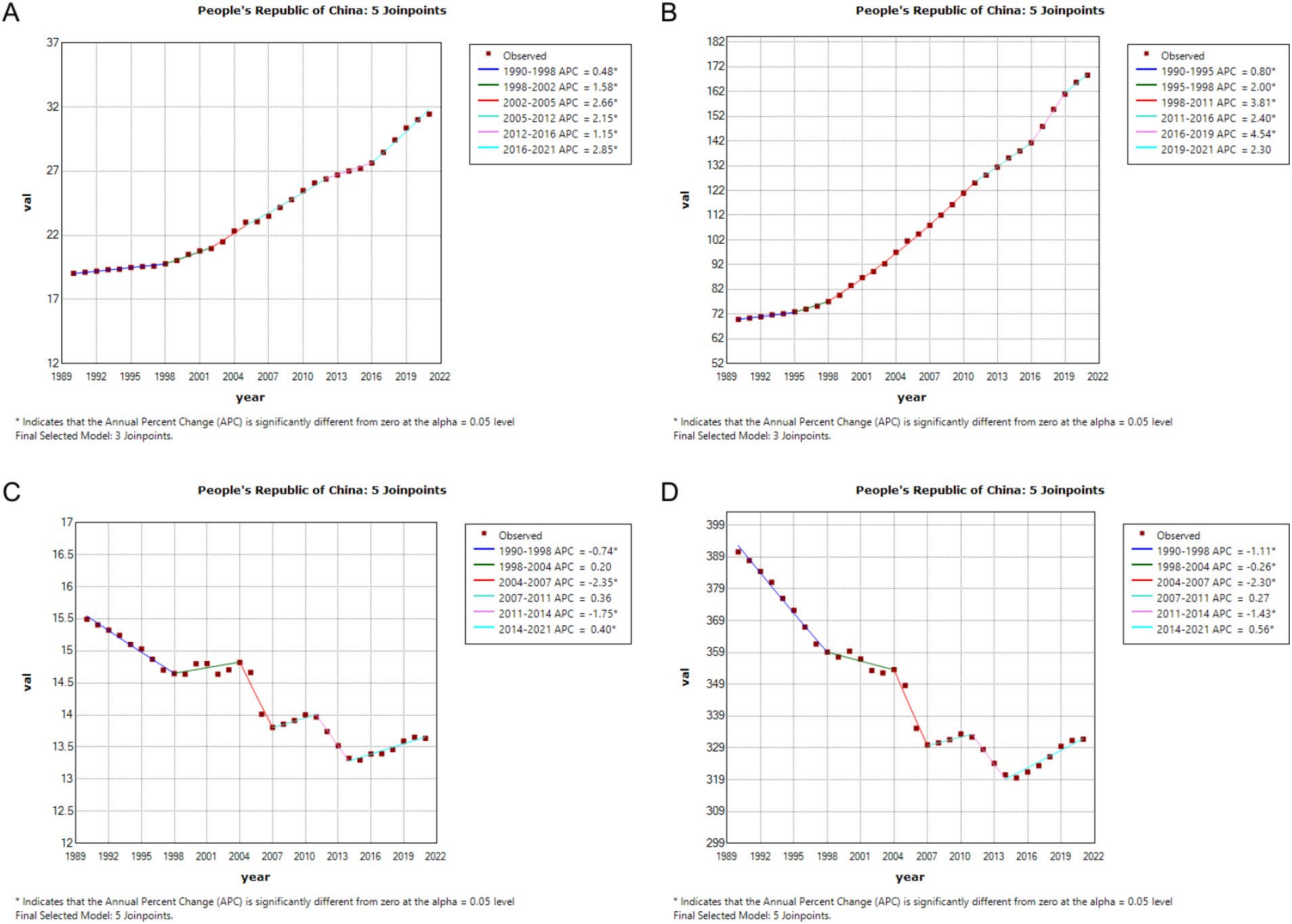
Age-Standardized Incidence Rate (ASIR), Age-Standardized Prevalence Rate (ASPR), Age-Standardized Mortality Rate (ASMR), and Disability-Adjusted Life Year Rate (ASDR) APCs for colorectal cancer in China, 1990–2021 (* indicates *p* < 0.05, denoting statistical significance). **(A)** ASIR; **(B)** ASPR; **(C)** ASMR; **(D)** ASDR.

**Figure 2:**
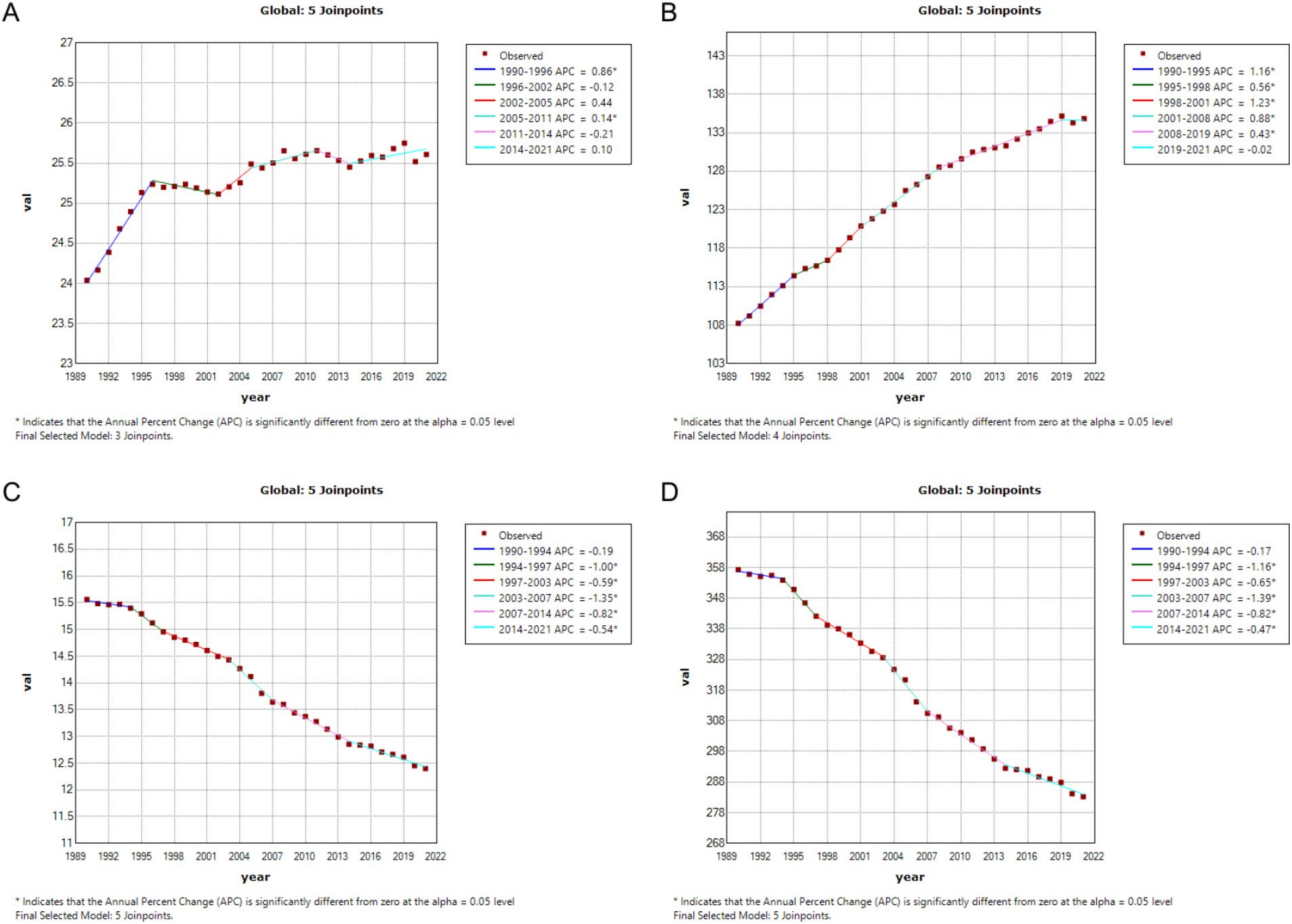
Age-Standardized Incidence Rate (ASIR), Age-Standardized Prevalence Rate (ASPR), Age-Standardized Mortality Rate (ASMR), and Disability-Adjusted Life Year Rate (ASDR) APCs for global colorectal cancer, 1990–2021 (* indicates *p* < 0.05, denoting statistical significance). **(A)** ASIR; **(B)** ASPR; **(C)** ASMR; **(D)** ASDR.

In China, the annual percentage change (APC) in CRC incidence was 1.58% during 1998–2002, which further increased to 2.85% during 2016–2021. In contrast, global CRC incidence remained nearly stable during 1996–2002 (APC = −0.12%), followed by a gradual increase during 2005–2011 (APC = 0.14%) and a slight rise during 2014–2021 (APC = 0.10%). Regarding prevalence, China experienced a notable acceleration in recent years, with an APC of 4.54% during 2016–2019. Globally, CRC prevalence increased from 108.25 per 100,000 in 1990 to 134.84 per 100,000 in 2021, with declining APCs in multiple periods, such as 0.43% during 2008–2019, and stabilization in 2021. Both mortality and DALYs demonstrated declining trends in China and globally. However, in China, mortality showed a slight rebound after 2014 (APC = 0.40%), whereas global mortality continued to decline during 2014–2021 (APC = −0.54%). Similarly, DALYs exhibited a decreasing trend in both China and globally. Overall, the increase in CRC incidence and prevalence was more pronounced in China compared to global trends, while the decline in mortality and DALYs was more significant globally. See Figures 1, 2 for detailed visualizations of these trends.

### 3.3 Colorectal cancer burden by age group in China and globally, 1990 and 2021

**Figure 3:**
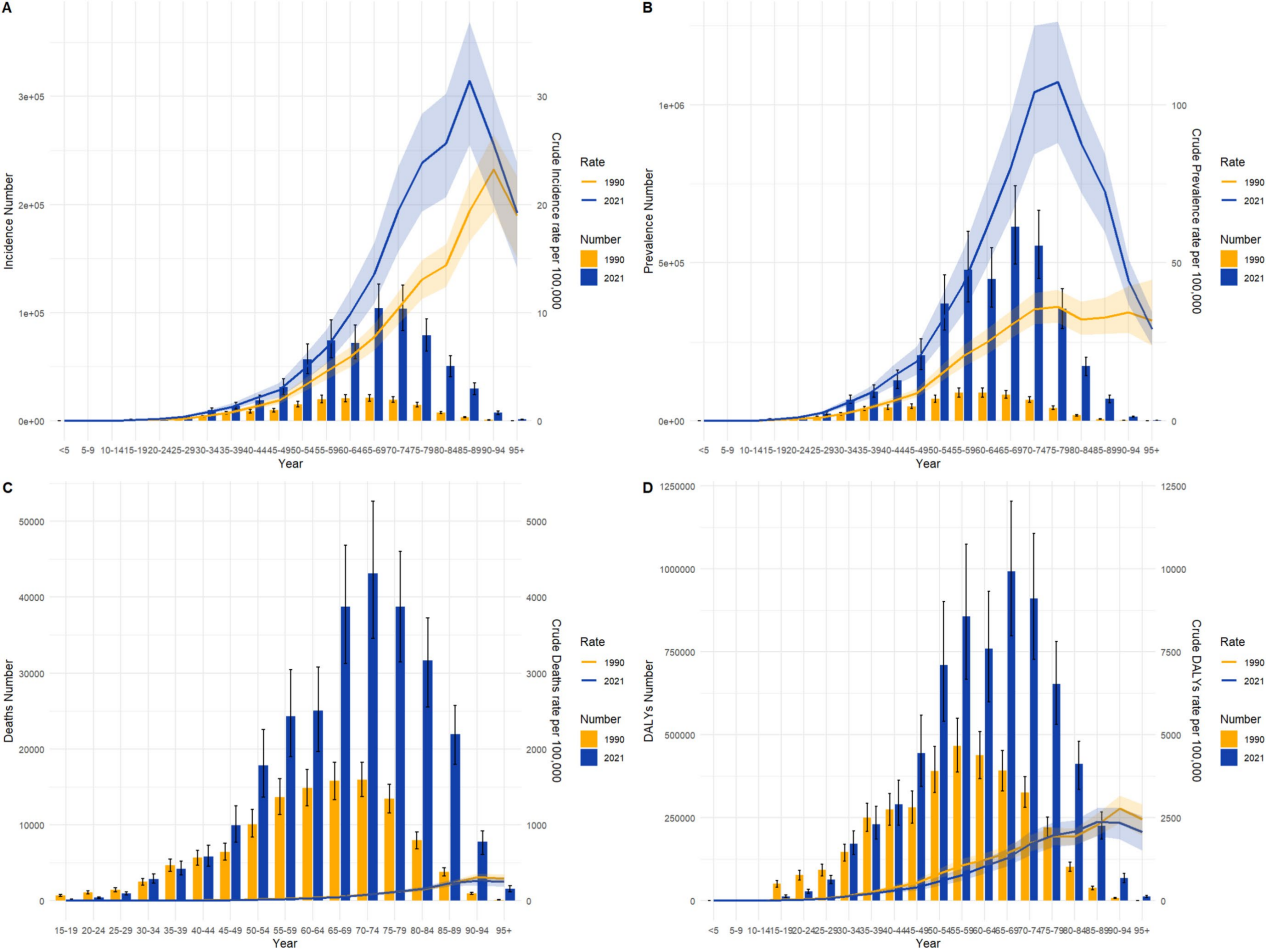
Comparison of age-specific incidence, prevalence, mortality, and DALYs counts and crude rates for colorectal cancer in China by age group in 1990 and 202. **(A)** Incidence and incidence rate. **(B)** Prevalence and prevalence rate. **(C)** Mortality and mortality rate. **(D)** Disability-Adjusted Life Years (DALY) and DALY rate.

**Figure 4:**
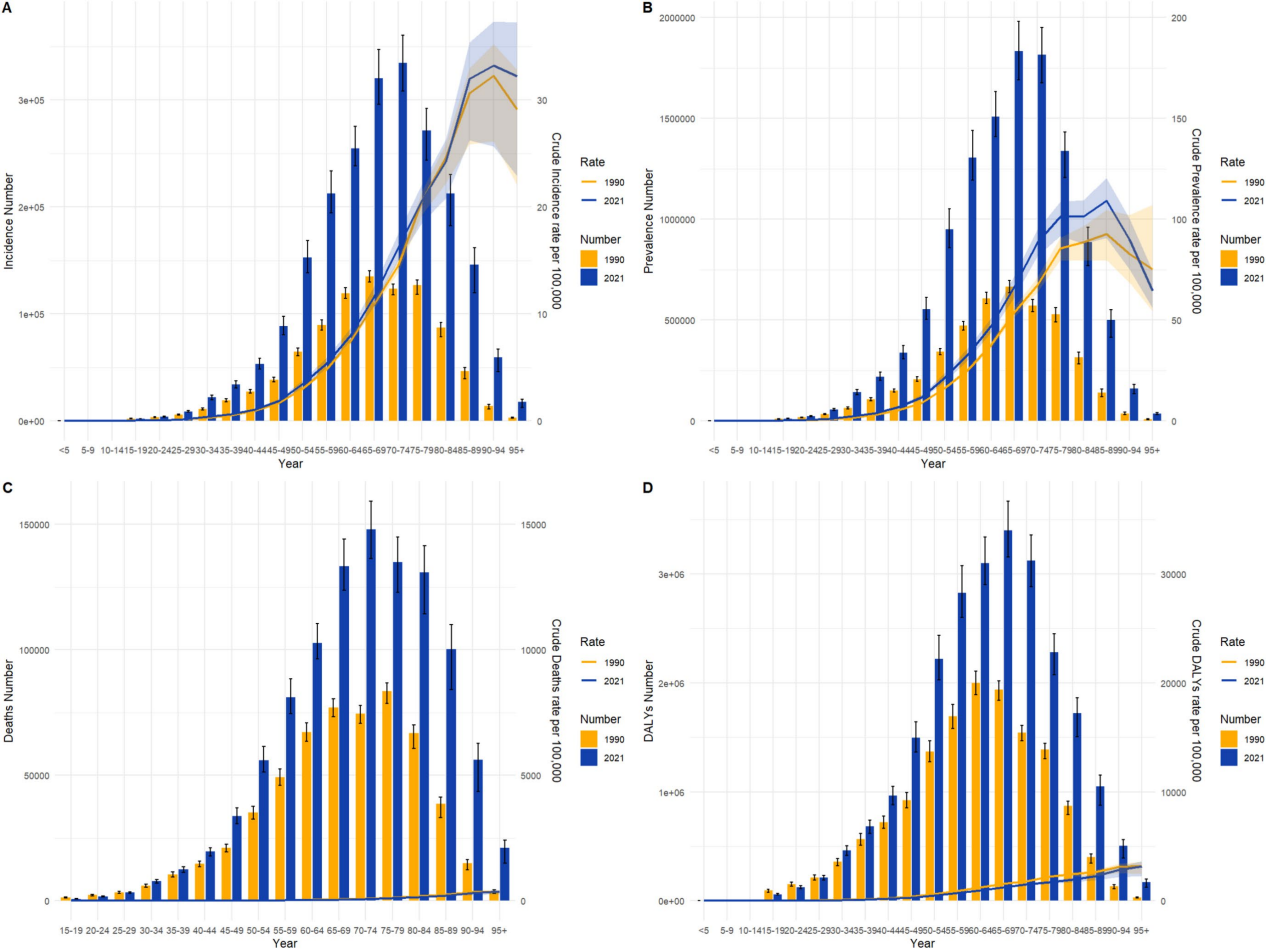
Comparison of age-specific incidence, prevalence, mortality, and DALYs counts and crude rates for colorectal cancer globally by age group in 1990 and 2021. **(A)** Incidence and incidence rate. **(B)** Prevalence and prevalence rate. **(C)** Mortality and mortality rate. **(D)** Disability-Adjusted Life Years (DALY) and DALY rate.

From 1990 to 2021, colorectal cancer incidence, prevalence, mortality, and DALYs across different age groups exhibited marked growth trends in China and globally, especially pronounced in middle-aged and older adult populations. While overall trends were similar, there were notable differences in specific distributions and growth rates. In terms of incidence, the number of cases and crude incidence rates in China in 2021 showed a significant increase among individuals aged 50 and above, with a peak in the 70–79 age group, and growth was relatively steady across age groups, while China demonstrated a steeper rise in older age groups, highlighting a sharper incidence increase in the older adult population. Prevalence trends reveal that China’s growth rate outpaced the global average, particularly among individuals over 50, with the crude prevalence rate for those aged 70–79 in China significantly exceeding global levels in 2021. Globally, prevalence numbers and rates showed a more even distribution, with stable growth across age groups, whereas China displayed a more pronounced upward trend, underscoring a higher prevalence rate in China’s older adult population. For mortality and DALYs, significant increases were observed for both China and globally among those aged 65 and above, though China’s mortality rate saw a notable resurgence in individuals over 80, while global mortality remained relatively stable. Globally, DALYs peaked in the 70–74 age group before gradually declining, while in China, DALYs reached a peak in the 75–79 age group and remained at high levels. See Figures 3, 4 for detailed comparisons.

### 3.4 Colorectal cancer burden by sex in China and globally, 1990 and 2021

**Figure 5:**
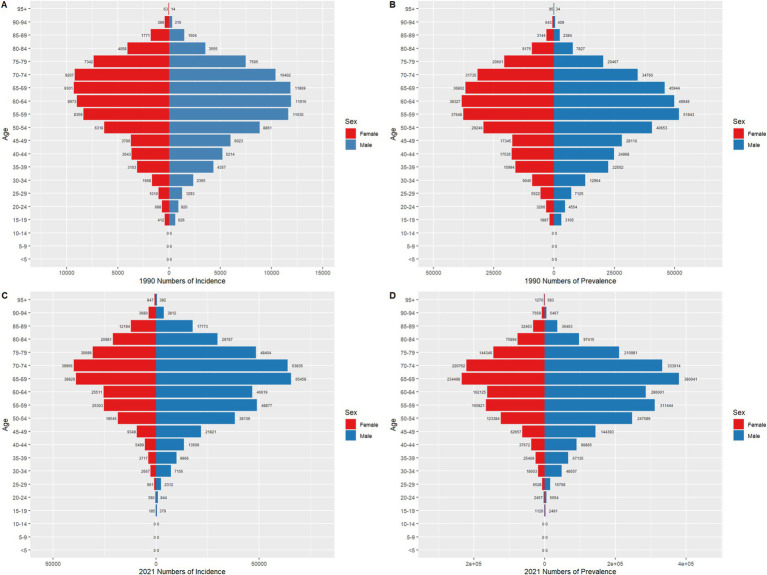
Comparison of age- and sex-specific incidence and prevalence counts for colorectal cancer in China in 1990 and 2021. **(A)** represents the number of colorectal cancer cases in China in 1990. **(B)** represents the number of prevalent colorectal cancer cases in China in 1990. **(C)** represents the number of colorectal cancer cases in China in 2021. **(D)** represents the number of prevalent colorectal cancer cases in China in 2021.

**Figure 6:**
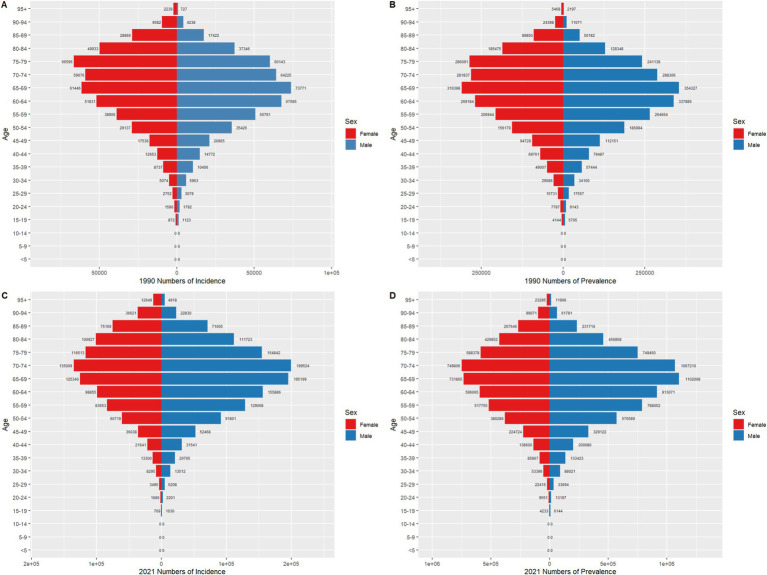
Comparison of age- and sex-specific incidence and prevalence counts for colorectal cancer globally in 1990 and 2021. **(A)** represents the number of colorectal cancer cases worldwide in 1990. **(B)** represents the number of prevalent colorectal cancer cases worldwide in 1990. **(C)** represents the number of colorectal cancer cases worldwide in 2021. **(D)** represents the number of prevalent colorectal cancer cases worldwide in 2021.

Between 1990 and 2021, the distribution of colorectal cancer incidence and prevalence by sex and age showed both shared trends and notable differences in China and globally. Additionally, sex disparities were pronounced, with incidence and prevalence rates for men generally higher than for women, particularly in those over 60, suggesting a heightened risk for colorectal cancer in men. However, the rate of increase in incidence and prevalence was markedly higher in China than globally, especially among the older adult. In 2021, the incidence and prevalence for the 70–79 age group in China showed a significant rise compared to 1990, while global figures remained relatively stable, with more uniform growth across age groups. Notably, the peak incidence age in China was centered in the 70–79 age group, whereas the global peak occurred in the 70–74 range, highlighting a trend toward older age at peak incidence in China. Although the male prevalence rate exceeded the female rate across all age groups in both China and globally, the sex disparity in older age groups was more pronounced in China by 2021. Specifically, among those aged 75 and older, Chinese males had a significantly higher disease burden than females, whereas the global sex difference in older adult groups was relatively smaller. See Figures 5, 6 for detailed comparisons.

### 3.5 Projected colorectal cancer burden by sex in China and globally over the next 15 years

**Figure 7:**
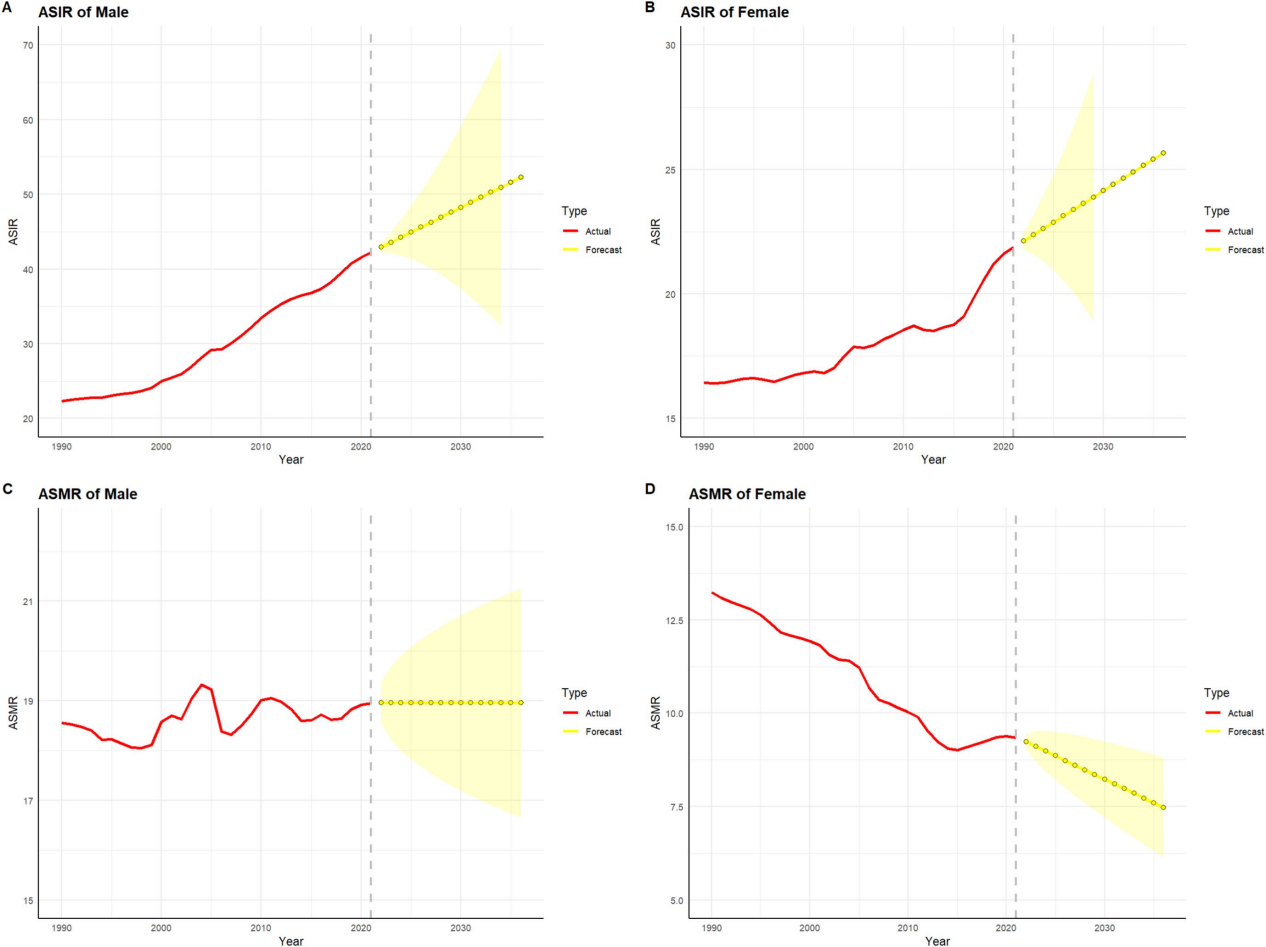
Projected trends in colorectal cancer incidence and mortality rates in China over the next 15 years (2022–2036). The red line represents the actual trend in colorectal cancer incidence and mortality rates in China from 1990 to 2021, while the yellow dashed line and shaded area denote the projected trend and its 95% confidence interval for 2022–2036. **(A)** represents the age-standardized incidence rate (ASIR) and its projection for colorectal cancer in Chinese males. **(B)** represents the age-standardized incidence rate (ASIR) and its projection for colorectal cancer in Chinese females. **(C)** represents the age-standardized mortality rate (ASMR) and its projection for colorectal cancer in Chinese males. **(D)** represents the age-standardized mortality rate (ASMR) and its projection for colorectal cancer in Chinese females.

**Figure 8:**
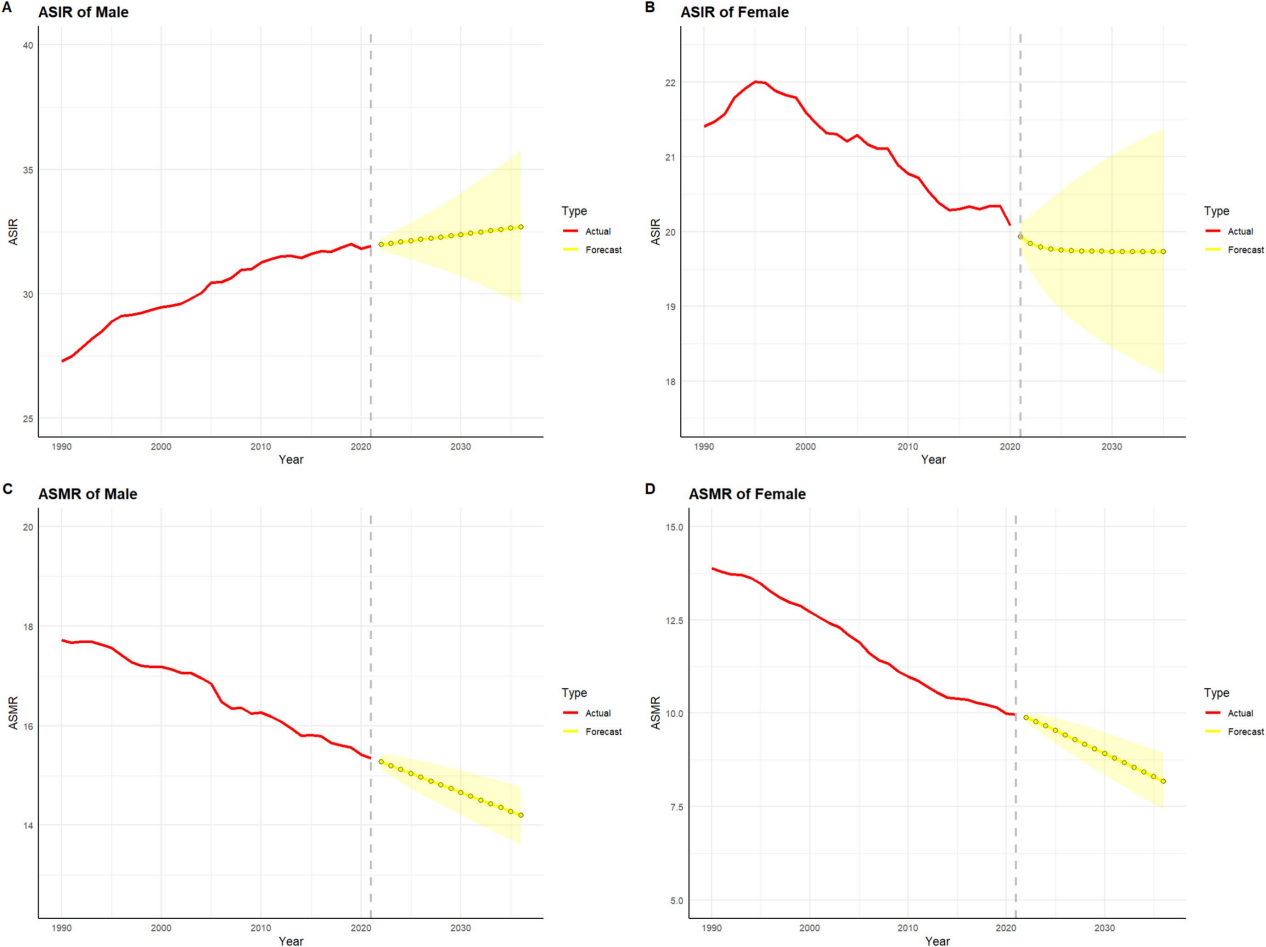
Projected trends in global colorectal cancer incidence and mortality rates over the next 15 years (2022–2036). The red line represents the actual trend in global colorectal cancer incidence and mortality rates from 1990 to 2021, while the yellow dashed line and shaded area indicate the projected trend and its 95% confidence interval for 2022–2036. **(A)** represents the age-standardized incidence rate (ASIR) and its projection for colorectal cancer in males worldwide. **(B)** represents the age-standardized incidence rate (ASIR) and its projection for colorectal cancer in females worldwide. **(C)** represents the age-standardized mortality rate (ASMR) and its projection for colorectal cancer in males worldwide. **(D)** represents the age-standardized mortality rate (ASMR) and its projection for colorectal cancer in females worldwide.

Figures 7, 8 present the historical trends and future projections of ASIR and ASMR for males and females in China and globally, respectively. Based on data analysis from 1990 to 2021 and projections for the next 15 years (up to 2036), distinct patterns and trends can be observed. In China, the ASIR for males (Figure 7A) is projected to remain at approximately 35 per 100,000 over the next 15 years. In contrast, the ASIR for females in China (Figure 7B) is expected to stabilize or even show a slight decline. Meanwhile, the ASMR for both males and females in China (Figures 7C,D) demonstrates a significant downward trend, which is projected to continue in the future. Globally, similar trends are observed, albeit with some differences in specific values. The ASIR for males worldwide (Figure 8A) is projected to exhibit a modest increase, while the ASIR for females (Figure 8B) is expected to remain around 17 per 100,000 over the next 15 years. Consistent with the trends in China, the ASMR for both males and females globally (Figures 8C,D) shows a sustained decline, which is anticipated to continue. A common feature in both China and globally is the significant decline in ASMR for males and females, despite variations in incidence rates.

## 4 Discussion

This study systematically analyzed the trends in the burden of colorectal cancer (CRC) in China and globally from 1990 to 2021, with projections extending to 2036. The results reveal that while the global burden of CRC has risen at a relatively slow pace, China is expected to face a more pronounced increase in the coming years, particularly among males and the older adult population. This trend provides critical insights for tailoring region-, gender-, and age-specific prevention and control strategies.

In terms of incidence, both the total number of cases and the ASIR have shown an upward trend from 1990 to 2021 in China and globally, with a steady increase expected to persist over the next 15 years. This phenomenon may be attributed to advancements in material living standards, which have led to changes in dietary patterns and lifestyle habits, as well as increased exposure to carcinogens, thereby facilitating the rise in colorectal cancer cases. Additionally, the widespread use of rectal exams, colonoscopy screenings, and a growing public awareness of the importance of regular health check-ups have contributed to this trend. However, the global trend exhibits more fluctuation compared to China, which could be explained by the large number of countries worldwide, the uneven levels of material living standards, and disparities in the development of health policies and healthcare systems. These factors contribute to the observed global variability. Furthermore, China’s AAPC is significantly higher than the global average, suggesting that unhealthy dietary and lifestyle patterns, coupled with advanced medical diagnostic capabilities, may be driving the higher AAPC of colorectal cancer in China from 1990 to 2021, which surpasses the global level.

Regarding prevalence, both the total number of cases and the ASPR have shown a steady upward trend from 1990 to 2021 in China and globally, with both exhibiting relatively smooth increases and no significant fluctuations. This phenomenon is closely linked to the increased exposure to carcinogenic factors and the accumulation of cases over time. As medical advancements continue and unhealthy lifestyle factors (21)—such as refined grain consumption, sedentary behavior, smoking, and alcohol use—become more prevalent, both China and the global population have seen a consistent rise in the number of cases. Additionally, China’s AAPC in ASPR is significantly higher than the global level, which may be attributed to improvements in medical care that have extended life expectancy for colorectal cancer patients. This has led to the continuous accumulation of colorectal cancer cases, resulting in the notable increase in the AAPC of ASPR in China from 1990 to 2021, which surpasses the global average.

In terms of mortality, both China and the global ASMR have shown a clear downward trend. Although the total number of deaths has increased due to population growth, the ASMR has continuously declined from 1990 to 2021, closely associated with the significant improvements in the diagnosis and treatment of colorectal cancer. In China, the ASMR has exhibited more fluctuation during this decline compared to the global trend, which may be linked to the rising incidence and prevalence of colorectal cancer. The increasing incidence and prevalence have led to a higher number of deaths, which somewhat offset the benefits brought about by advancements in medical care, resulting in noticeable fluctuations in the ASMR decline. Comparatively, the AAPC difference in ASMR between China and the global trend from 1990 to 2021 is minimal, indirectly highlighting the rapid progress and high standard of medical care in China. Projections indicate that both China and the global ASMR will continue to decrease over the next 15 years, reinforcing the sustained trend of improving healthcare outcomes in the future.

In terms of DALYs, both Chinese and global ASDR have shown a distinct downward trend. While the total number of DALYs has increased due to population growth, the ASDR has steadily declined from 1990 to 2021, closely linked to advancements in healthcare that have reduced the impact of colorectal cancer on life expectancy. In China, the ASDR has exhibited more fluctuation during this decline compared to the global trend, which may be attributed to the rising incidence and prevalence of colorectal cancer. The increased incidence and prevalence have led to greater life expectancy losses, which have partially offset the medical benefits brought about by healthcare improvements. However, the difference in the AAPC in ASDR between China and the global trend from 1990 to 2021 is not significant, highlighting the substantial progress in China’s healthcare system. This progress has effectively mitigated the negative impact of the rising incidence and prevalence on life expectancy, significantly reducing the overall disease burden of colorectal cancer in China.

The findings are expected to guide policymakers and healthcare professionals in devising targeted interventions including screening to mitigate future CRC burden, ultimately improving health outcomes and reducing healthcare costs both nationally and globally.

In China, CRC screening has been integrated into the national major public health service programs, namely the “Urban Cancer Early Diagnosis and Treatment Project” and the “Rural Cancer Early Diagnosis and Treatment Project,” since 2012, covering multiple provinces and cities across the country. Pilot CRC screening initiatives were first implemented in economically developed coastal regions in the east (e.g., Shanghai, Zhejiang, and Jiangsu) and have since been gradually expanded to central and western regions. In some areas, CRC screening costs have been included in medical insurance reimbursement schemes, alleviating the financial burden on residents. As of 2021, the national CRC screening coverage rate reached approximately 30%, although coverage remains lower in rural and economically underdeveloped regions. Through the implementation of these policies, significant progress has been made in CRC screening efforts in China. To address the significant burden of colorectal cancer in China, it is imperative to strengthen colorectal cancer screening efforts, aiming for comprehensive coverage across both urban and rural areas. Increasing the frequency of screening during routine health examinations and ensuring a systematic transition from digital rectal examination to colonoscopy-based screening will be crucial in mitigating the escalating burden of colorectal cancer.

This study has several strengths. The data coverage is comprehensive with the use of data from the GBD database (1990–2021). This study covers an extended period and a wide range of countries and regions, enabling a comprehensive reflection of CRC burden trends in China and globally. The consistency and comparability of the data sources enhance the reliability of the analysis. Furthermore, this study utilizes multidimensional indicator analysis including indicators such as ASIR, ASPR, ASMR, and DALYs. These indicators were used to assess CRC from multiple angles, providing policymakers with comprehensive references. Lastly, this study also provides prospective trend projection through the use of the ARIMA model to project for the years 2022–2036, meaning this study not only presents current CRC burden trends but also reasonably predicts future burdens, offering forward-looking guidance for CRC prevention and control strategies.

Nevertheless, this study has some limitations. Although the GBD database provides extensive coverage, the quality of some data may be subject to localized data bias, particularly due to inconsistencies in the diagnosis, recording, and reporting of colorectal cancer worldwide, which could lead to an underestimation of the disease burden. Additionally, as the database integrates data from multiple sources, variations in the timeliness and frequency of updates across regions may affect the accuracy of the data. Furthermore, The ARIMA model used in this study relies on historical data for projections which could lead to uncertainty in model predictions. However, future changes in social, economic, and healthcare conditions, as well as disease control measures, may influence the actual burden of colorectal cancer. Consequently, the predictive results carry a degree of uncertainty, particularly for long-term projections, where these factors could introduce potential biases. Lastly, the analysis is based on macro-level data and does not fully account for individual-level risk factors such as dietary habits, lifestyle, and occupational exposures, nor does it consider environmental influences such as air pollution and sanitation. As a result, the findings may have limitations when applied to specific subpopulations.

## 5 Conclusion

This study analyzed the trends in the burden of colorectal cancer in China and globally from 1990 to 2021, with projections extending to 2036. The findings reveal a steady increase in the global burden of colorectal cancer, with a more pronounced acceleration observed in China, particularly among males and the older adult population. These results provide a robust scientific foundation for the development of targeted prevention and control strategies worldwide and in China, highlighting the necessity of implementing precise and evidence-based interventions to address this growing public health challenge. To address the significant burden of colorectal cancer in China, it is imperative to strengthen colorectal cancer screening efforts, aiming for comprehensive coverage across both urban and rural areas. Increasing the frequency of screening during routine health examinations and ensuring a systematic transition from digital rectal examination to colonoscopy-based screening will be crucial in mitigating the escalating burden of colorectal cancer.

## Data Availability

The original contributions presented in the study are included in the article/supplementary material, further inquiries can be directed to the corresponding author.

## Footnotes

^1^ https://ghdx.healthdata.org/gbd-2021
