# Correction: Analysis of trends in the burden of colorectal cancer in China and globally from 1990 to 2021 with projections for the next 15 years: a cross-sectional study based on the GBD database

**DOI:** 10.3389/fpubh.2025.1635228

**Published:** 2025-06-27

**Authors:** Yulai Yin, Xiaoyu Zhang

**Affiliations:** ^1^Cangzhou Central Hospital Affiliated to Hebei Medical University, Cangzhou, China; ^2^Department of Thyroid and Breast Surgery, Cangzhou Central Hospital, Cangzhou, China

**Keywords:** colorectal cancer, disease burden, incidence rate, prevalence rate, prevention

In the published article, there was an error in **affiliations** for author Yulai Yin. Instead of

“1. Nankai University School of Medicine, Nankai University, Tianjin, China
2. Graduate School, Tianjin University of Traditional Chinese Medicine, Tianjin, China
3. Department of General Surgery, The First Affiliated Hospital of Jinzhou Medical University, Jinzhou, China
4. Department of Colorectal Surgery, Tianjin Union Medical Center, The First Affiliated Hospital of Nankai University, Tianjin, China”,

it should be

“^1^Cangzhou Central Hospital Affiliated to Hebei Medical University, Cangzhou, China”.

The authors apologize for this error and state that this does not change the scientific conclusions of the article in any way. The original article has been updated.

